# Research on motion planning for an indoor spray arm based on an improved potential field method

**DOI:** 10.1371/journal.pone.0226912

**Published:** 2020-01-10

**Authors:** Dongjie Zhao, Bin Zhang, Ying Zhao, Qun Sun, Chuanjun Li, Chong Wang

**Affiliations:** 1 School of Mechanical & Automotive Engineering, Liaocheng University, Liaocheng, China; 2 College of Engineering, China Agricultural University, Beijing, China; Nanyang Technological University, SINGAPORE

## Abstract

The target spraying effect of spray robots mainly depends on the control performance of the spraying arm during the processes of aiming and tracking. To further improve the robustness of the endpoint control and positioning accuracy of the spray arm, an improved potential field algorithm for the motion planning and control of the spray arm is proposed based on prophase research. The algorithm introduces a velocity potential field, visual field constraints and joint position limit constraints into the traditional artificial potential field method. The velocity potential field is used to ensure that the target state of the spraying arm is at the same velocity as the target crop (relative velocity) to achieve stable target tracking. The visual field constraints and joint position limit constraints are utilized to ensure the efficiency of the visual servo control and the movement of the spray arm. The algorithm can plan a feasible trajectory for the spraying arm in Cartesian space and image space, and use the speed controller to control the spraying arm movement along the trajectory for aiming and tracking. Simulation analysis shows that the algorithm can plan better motion trajectories than the servo controller based on image moments in previous studies. In addition, the experimental results show that the algorithm can effectively improve the robustness of targeting and tracking control for the spray robot.

## Introduction

For crops planted in a greenhouse environment with large plant spacing, such as cucurbit seedlings and lettuce seedlings in the early growing season, it is important to use target spraying technology to apply fertilizers and pesticides according to their position and size. In addition, it is of significance to improve the utilization rate of medicinal liquids and to alleviate soil pollution. Introducing a visual servo control into the spraying robot system and using the visual information obtained from the visual sensor to guide the spraying arm to complete the target movement, can satisfy the accuracy requirements of target operation and improve the adaptability of the spraying robot in unstructured environments (e.g., greenhouses) [1–5].

It was found in reference [6] that spray robots can achieve a good target effect by using the visual tracking method based on image moments and a hybrid vision system that includes a monocular scene camera and a monocular (or multi) eye-in-hand camera. However, for the servo control of the spray arm, when the initial pose and the desired pose are quite different, the motion trajectory planned only by the speed controller is poor, which limits the speed of the spraying robot. Furthermore, the vibration of the spray robot caused by occasional larger undulations or obstacles on smooth greenhouse pavement will cause target crops to leave the field of view of the eye-in-hand camera, and the servo process will sometimes fail.

Meanwhile, the joint manipulator has a faster response speed and a more irregular workspace than the Cartesian-coordinate manipulator, and its irregular workspace has some restrictions on the motion trajectory of the manipulator [7–8]. Therefore, it is necessary to optimize the visual servo process by adding various constraints, and applying the path planning method to reasonably control the motion of the manipulator.

The common methods in the trajectory planning include genetic algorithm [9–10], simulated annealing [11–12], artificial neural network [13–14], A* algorithm [15], vector field method [16], adaptive algorithm [17–18], particle swarm optimization algorithm [19–20], artificial potential field method [21–22], etc. Among these algorithms, the artificial potential field method has a simple structure, is convenient for real-time control on hardware entities, and can usually plan smoother and safer paths. This method has been widely used in real-time obstacle avoidance and smooth trajectory control [23–25]. However, the algorithm also has some shortcomings, such as goal nonreachable with obstacle nearby (abbr. GNRON) and an insufficient dynamic path planning ability [26], which can be improved together with specific problems.

In this paper, the traditional artificial potential field method is appropriately improved according to visual tracking and target requirements of the spray arm, and the improved algorithm is used to plan the motion trajectory of the spray arm under the conditions of visual field constraints and joint limit constraints. The effect is verified by simulation and prototype tests.

## Target spray arm and target operation

### System scheme for target spray robot

**Fig 1:**
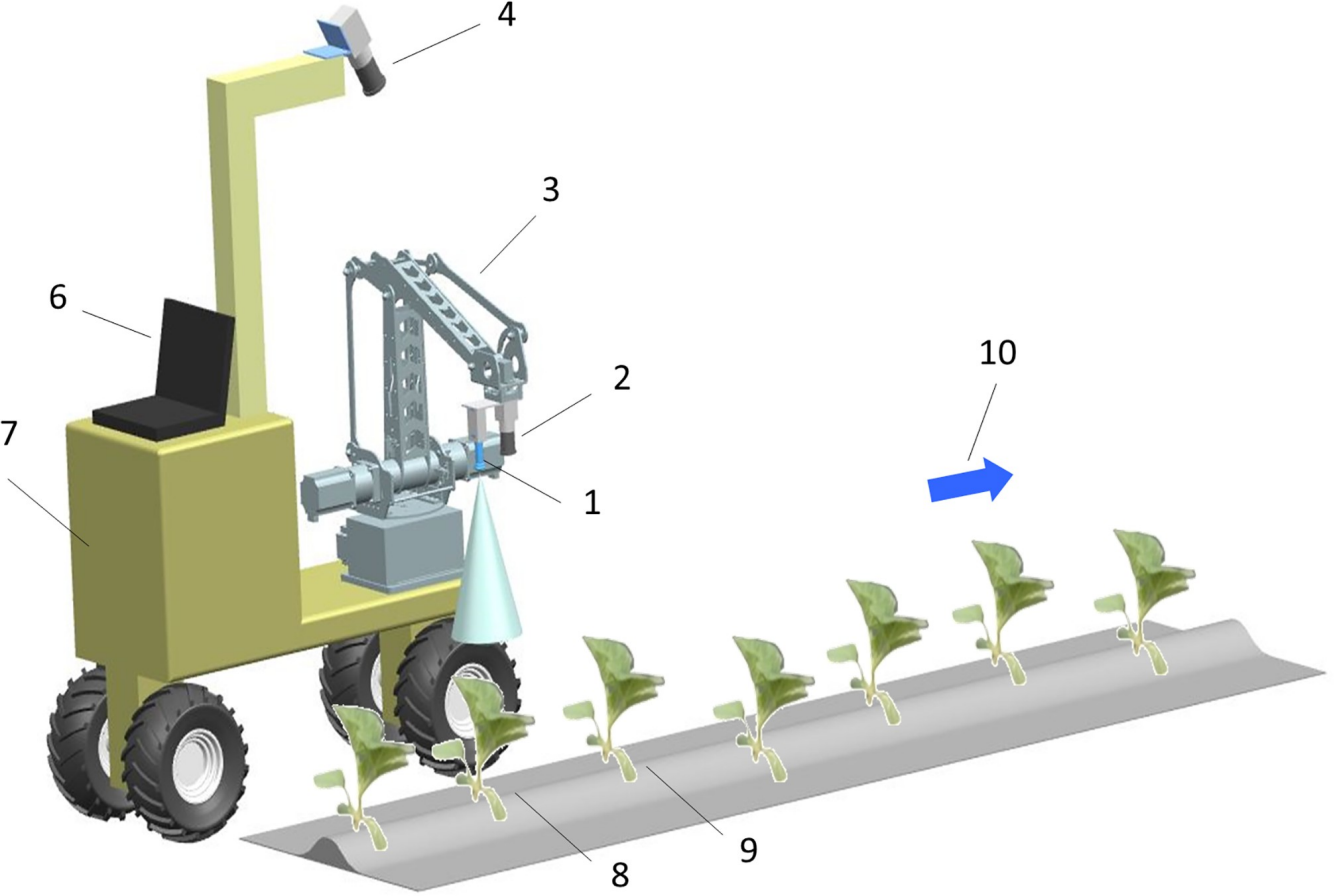
Target spraying robot system. 1. Nozzle 2. Eye-in-hand camera 3. Spray arm 4. Scene camera 5. Slave computer (DSP, not shown) 6. Host computer (PC) 7. Movement platform 8. Target crop 9. Object crop 10. Robot’s motion direction.

A hybrid vision structure similar to that in reference [6], including one scene camera and one (or several) eye-in-hand camera, is adopted by the target spray robot (Fig 1). The scene camera, mounted in the front of the robot body, is used to obtain location information about the object crops. The eye-in-hand camera, mounted at the end of the spray arm with the nozzle, is used to provide precise localization of the target crop. The control system consists of one personal computer (abbr. PC, host computer) and one(or several) digital signal processor (abbr. DSP, slave computer). The PC generates and transmits control instructions to the DSP by analyzing the scene camera images and real-time state information of the working parts (spray arm, nozzle, etc.) provided by the DSP. The DSP controls the movement of the spray arm and nozzle according to the PC instructions, the eye-in-hand camera images and the other sensors.

A ZUTO460 4-DOF series manipulator is selected to be the spray arm. The manipulator can achieve the movement along X-axis, Y-axis, Z-axis and the rotation along Z-axis of the end-effector, which meets the requirements of the translational motion of the nozzle in vertical spray mode, and is suitable for fertilization and pesticide application for many greenhouse crops. Furthermore, compared with the Cartesian coordinate manipulator in reference [6], the manipulator has a larger working space and a faster moving speed, and thus, more easily meets the target requirements when the robot moves more quickly.

The process of the spray arm guiding the nozzle to a target spray along with the forward motion of the spray robot can be described as follows: first, the control system, according to the position information about the object crops provided by the scene camera, controls the spray arm movement to make the crops enter smoothly into the eye-in-hand camera’s view. Then, according to precise information about the target crop provided by the eye-in-hand camera, the control system controls the spray arm to move the nozzle quickly into spray position (target) and ensures the relative position between the nozzle and the target crop is unchanged (tracking spray). It is easy to understand that the effect of target spray is mainly determined by the visual servo control accuracy of the spray arm during the targeting and tracking stage.

### Description of target operation issue

The spray arm needs to guide the nozzle to fulfill target and tracking spray operations during the forward movement of the spray robot. Regarding path planning, the motion of the nozzle during the targeting stage can be regarded as point to point movement, and it is necessary to ensure that the trajectory of the nozzle is smooth, and the path is short. The motion of the nozzle during the tracking spray stage can be regarded as continuous path movement, so that the nozzle must stably and accurately track the motion trajectories of the target crop relative to the spray robot. Moreover, to avoid the failure of the visual servo, the target crop should always be in the field of the eye-in-hand camera, and the planned targeting and tracking trajectories need to satisfy the spray arm’s workspace, which is an irregular polyhedron due to structural constraints.

To solve above problems, a path-planning and visual tracking method based on an improved artificial potential field is proposed. The basic idea is that, based on the traditional artificial potential field, velocity potential field is introduced to meet the needs of stable tracking; in addition, field of view constraint and joint position limit constraints are introduced to ensure the validity of the servo control and the motion of the spray arm. Thus, a feasible trajectory is planned in Cartesian space and image space, and the image-based visual servo method is used to track the trajectory.

## Trajectory planning and target control based on the improved potential field method

### Traditional artificial potential field method

The traditional artificial potential field method regards the motion of a robot in planned space as a kind of motion in a virtual force field. Target points attract the robot, while obstacles or threat areas repel it, and it moves toward the target points under the action of a composition force. The traditional gravitational field U_*att*_ and the repulsive force field U_*rep*_ are usually defined as

$$U_{att}(P)=0.5\alpha {\left|P_{t}-P\right|}^{2}$$ (1)

$${\text{U}}_{reP}\left(P\right)=\{\begin{array}{ll} 0.5\beta {\left(\frac{1}{\left|P_{o}-P\right|}-\frac{1}{\rho_{0}}\right)}^{2}, & if\left|P_{o}-P\right|<\rho_{0}\\ 0, & if\left|P_{o}-p\right|\geq \rho_{0} \end{array}$$ (2)

where

***P***——current position vector of the robot

***P***_*t*_——position vector of a target point

***P***_*o*_——position vector of the closest point between an obstacle and the robot

*ρ*_0_——influence distance of the obstacle repulsion field, *mm*

*α*, *β*——positive proportional gain coefficient of gravity and repulsion

Gravity ***F***_*att*_ and repulsion ***F***_*rep*_ are equal to the negative gradients of U_*att*_ and U_*rep*_ respectively, while the virtual force on the robot ***F*** is the sum of the two vectors.

$$\left(P)=F_{att}(P)+F_{rep}(P)=-\nabla U_{att}(P)-\nabla U_{rep}(P\right)$$ (3)

where

∇——Laplacian operator

The artificial potential field method can iteratively plan the discrete trajectory of the robot. The virtual force F determines the motion direction of the robot in the next time step:

$$P_{k+1}=P_{k}+\varepsilon \frac{F(P)}{\left|F(P)\right|}$$ (4)

where

***P***_***k***_——current position vector of the robot

***P***_**k+1**_——current position vector of the robot in the next time step

***ε***——iterative step size

### Improved method

During the process of targeting and tracking spray, the spray robot keeps moving forward, which is identical to the situation in which the robot remains still and the target crop keeps moving back, that is, the situation in which the target is moving. Since the traditional artificial potential field model does not consider the speed factor, the planned path is prone to oscillation when tracking moving targets. That is, this method can only achieve a fast approach to moving targets, but it is difficult to stably track objects. Therefore, a velocity term is introduced into the gravitational field function to make the target state of the spray arm move at the same speed as the target crop.

Although greenhouse pavement is relatively flat, there are still occasional large fluctuations or obstacles, which may cause the target crop to escape the field of view of the hand-eye camera, and the servo process to fail in some cases. Therefore, field of view constraints are introduced, that is, the four boundaries of the field of view are regarded as obstacles. In addition, due to structural constraints, the joints of the waist and arm of the spray arm can only be rotated in specific ranges, and the limit positions on both sides of the ranges should also be regarded as obstacles.

The above gravitational and repulsive terms are defined in different description spaces. In particular, the four boundaries of the field of view are defined in image space, the limit positions of the joints are defined in joint space, the velocity term of the gravitational field is defined in Cartesian space, and its location term is defined in image space (according to reference [6], the expected spray position of the target crop is determined by its image moment). If the artificial potential field method is used to plan the trajectory, the virtual forces should be transformed into the same description space. In this paper, the method described in reference [27] is applied to transform the virtual forces between different descriptive spaces. Thus, if the potential field function U_*f*_ = U(*f*(***P***)), and *f*(***P***) is continuously differentiable in the feasible region of ***P***, then the virtual force ***F***_***f***_ can be expressed as

$$F_{f}(P)=-(\frac{\partial f}{\partial P}{)}^{+}\nabla U_{f}$$ (5)

where

$(\frac{\partial f}{\partial P}{)}^{+}$ —— the inverse (or pseudo inverse) of partial derivative $\frac{\partial f}{\partial P}$

On the premise of satisfying the field of view and joint limit constraints, the improved artificial potential field method is applied to plan the discrete trajectory of the camera in Cartesian space, which then is mapped into image space and tracked with the image-based visual servo controller.

### Gravitational potential field and gravitation

To stably track the target crop and avoid oscillation, a velocity term is introduced into the gravitational potential field, and the gravitational potential field function is defined as

$$U_{att}(s,v)=0.5\alpha_{1}{\left|s^{*}-s\right|}^{2}{+0.5\alpha }_{2}{\left|v_{t}-v\right|}^{2}$$ (6)

where

s——image moment vector of the target crop $\left(\frac{m_{10}}{m_{00}},\frac{m_{01}}{m_{00}},k\sqrt{\frac{\mu_{20}+\mu_{02}}{m_{00}}}\right)$, (pixel, pixel, pixel)

s*——expected value of s, (pixel, pixel, pixel)

v_t_——relative velocity of the target crop, mm/s

v——velocity of the end of the spray arm(or the eye-in-hand camera), mm/s

*α*_1_, α_2_——positive proportional gain coefficient

The negative gradients of U_*att*_(***s***, ***v***) relative to ***s*** and ***v*** can be called the relative position gravity ***F***_*atts*_ and the relative velocity gravity ***F***_*attv*_, respectively, and

$$F_{atts}(P)=-\nabla_{s}U_{att}(s,v)=\alpha_{1}L_{s}^{+}(s^{*}-s)$$ (7)

$$F_{attv}(P)=-\nabla_{v}U_{att}(s,v)=\alpha_{2}(v_{t}-v)$$ (8)

$$F_{att}(P)=F_{atts}{(P)+F}_{attv}(P)$$ (9)

where

$L_{s}^{+}$——inverse matrix of the image Jacobian matrix related to ***s***

***F***_*att*_(***P***)**—**—virtual resultant gravity

The function of F_atts_(P) makes the spray arm move along the shortest path to the desired spray position, its direction is from the current position to the desired position and its size is proportional to the relative position. The function of ***F***_*attv*_(***P***) makes the target state of the spray arm move at the same speed as the target crop; its direction is the motion direction of the target crop relative to the spray arm, and is proportional to the relative velocity. When the spray arm is aligned with the desired spray location and the relative velocity between the spray arm and the target crop is 0, ***F***_*att*_(***P***) = 0.

### Repulsion field and repulsion force of field of view constraint

**Fig 2:**
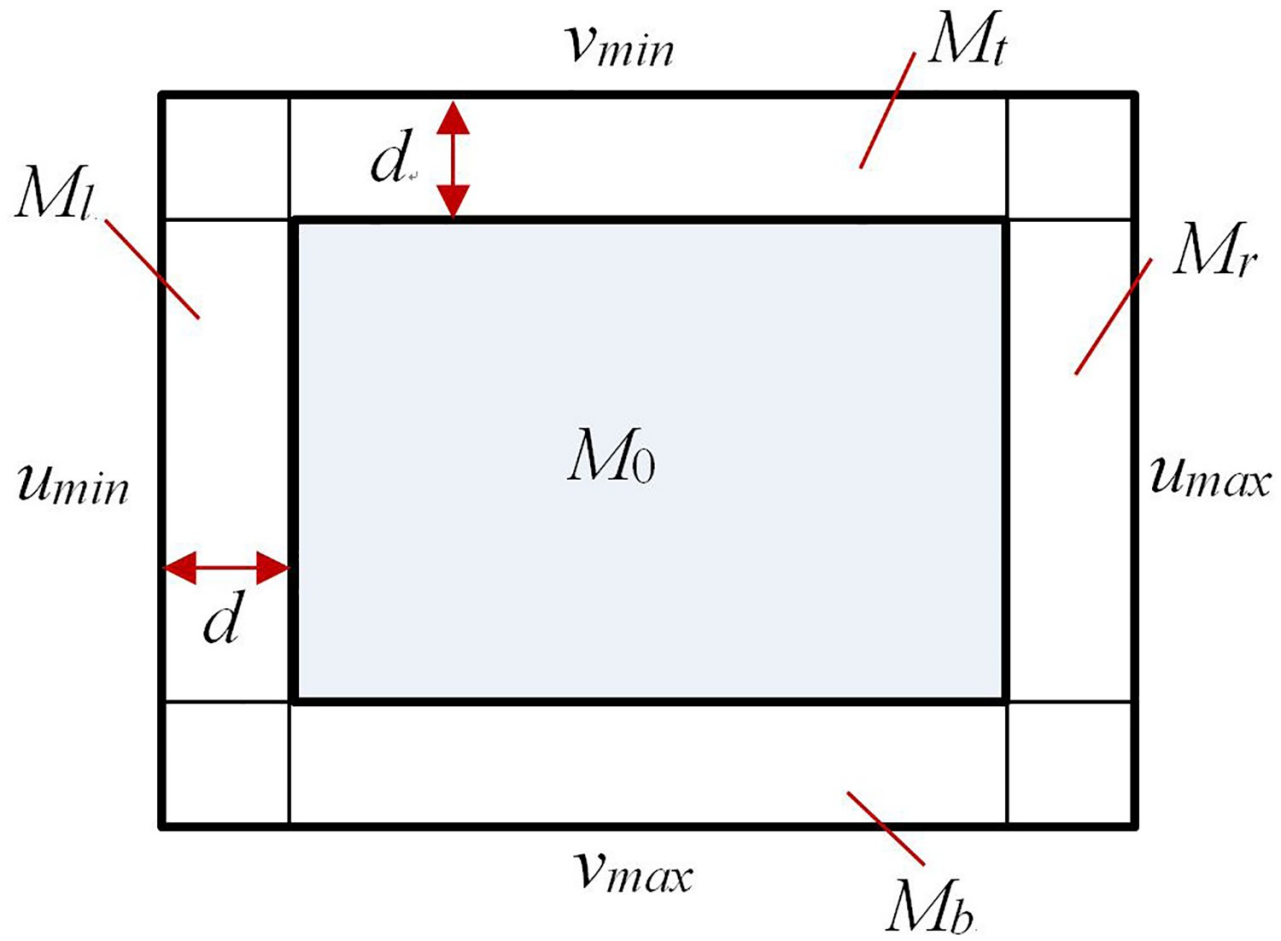
The visual field of the eye-in-hand camera.

Suppose the crop image will be kept in the camera’s view while the centroid of the crop image k_0_(u_0_, v_0_) is in region M of the camera’s view (M’s boundary is u_min_, u_max_, v_min_, v_max_). Let d be the influence distance of the boundary of the field of view (Fig 2).

**Fig 3:**
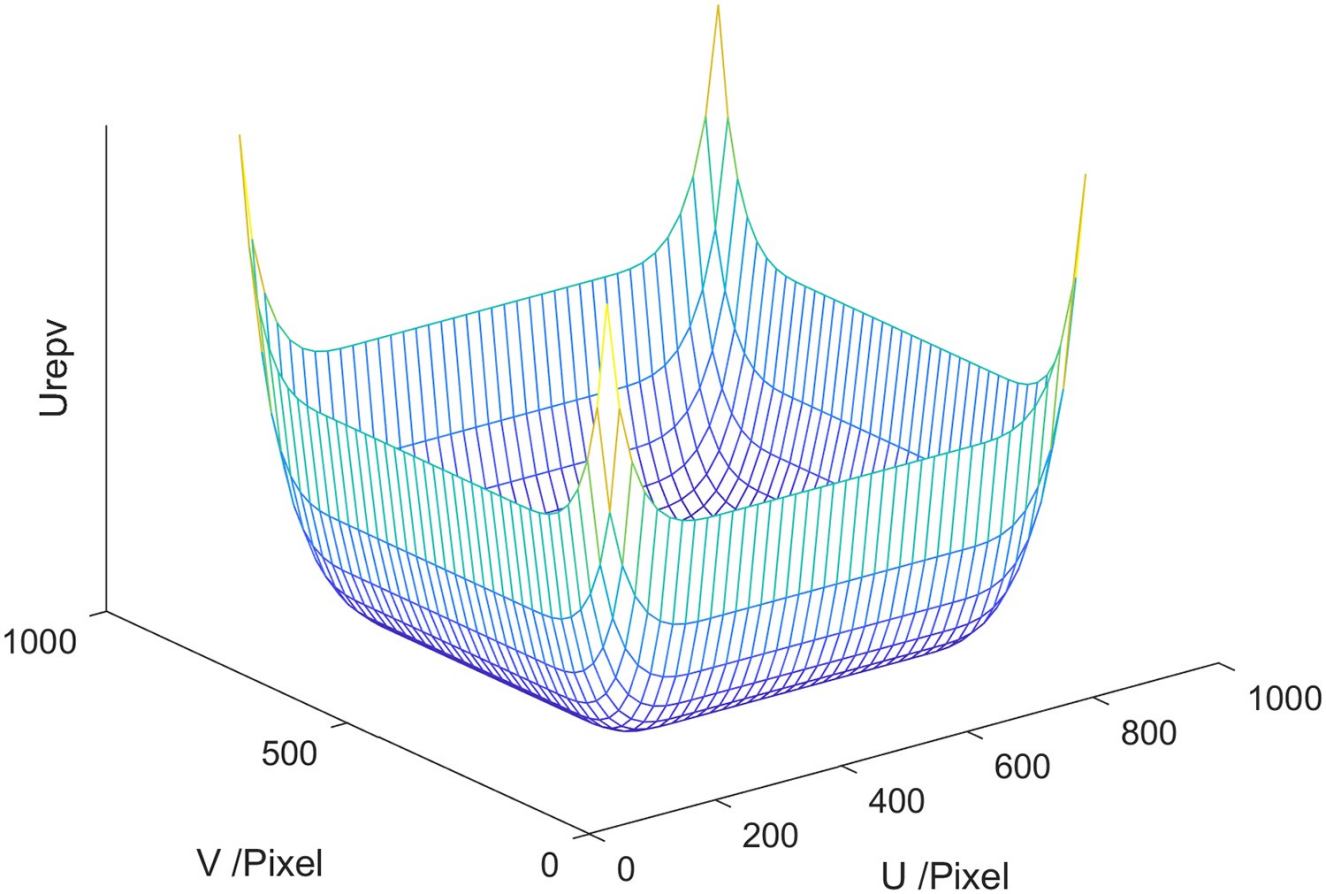
The repulsion field of the visual field constraint.

To prevent k_0_ from escaping from M, k_0_ should be repulsed to return to safe region M_0_ when it moves in M_l_, M_r_, M_t_ and M_b_. By introducing logarithmic terms into the repulsive field function, the repulsive force tends to be infinite when *k*_0_ approaches the boundary of the camera’s view. The repulsion field function is (Fig 3):

$$U_{repv}(k_{0})=U_{repv}(u_{0}){+U}_{repv}(v_{0})$$ (10)

where

$$U_{repv}\left(u_{0}\right)=\{\begin{array}{ll} -{\left(u_{0}-d-u_{min}\right)}^{2}\text{ln}\left(\frac{u_{0}-u_{min}}{d}\right), & if\;u_{0}\in \left(u_{min},u_{min}+d\right)\\ -{\left(u_{0}+d-u_{max}\right)}^{2}\text{ln}\left(\frac{u_{max}-u_{0}}{d}\right), & if\;u_{0}\in \left(u_{max}-d,u_{max}\right)\\ 0, & if\;u_{0}\in \left[u_{min}+d,u_{max}-d\right] \end{array}$$

$$U_{repv}\left(v_{0}\right)=\{\begin{array}{ll} -{\left(v_{0}-d-v_{min}\right)}^{2}\text{ln}\left(\frac{v_{0}-v_{min}}{d}\right), & if\;v_{0}\in \left(v_{min},v_{min}+d\right)\\ -{\left(v_{0}+d-v_{max}\right)}^{2}\text{ln}\left(\frac{v_{max}-v_{0}}{d}\right), & if\;v_{0}\in \left(v_{max}-d,v_{max}\right)\\ 0, & if\;v_{0}\in \left[v_{min}+d,v_{max}-d\right] \end{array}$$

**Fig 4:**
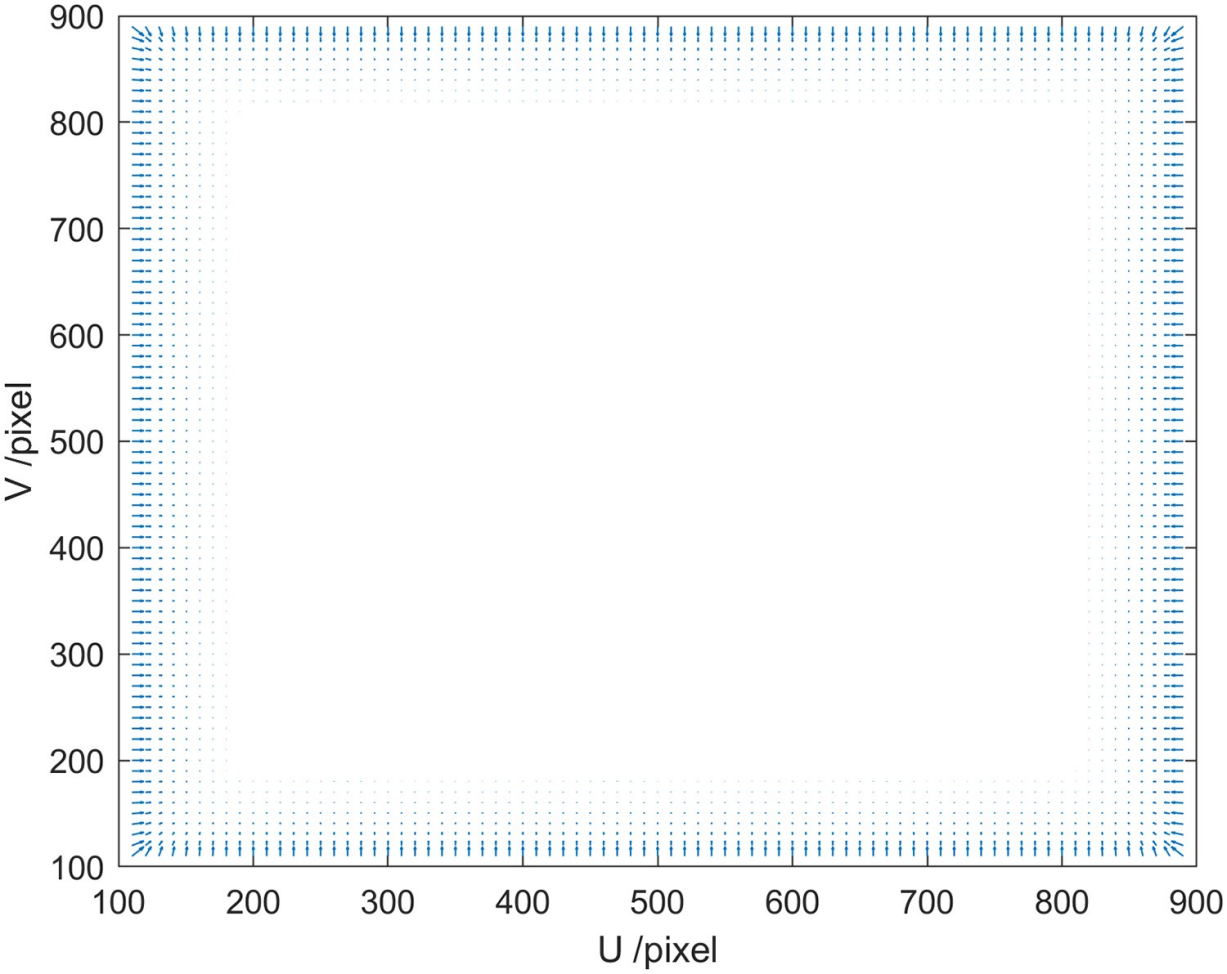
The repulsive force of the visual field constraint.

The repulsive force ***F***_*repv*_ (as shown in Fig 4, the length and direction of the arrow represents the magnitude and direction of the repulsion force, respectively) of the field of view of the spray arm can be expressed as

$$F_{repv}=\{\begin{array}{ll} L^{+}{\left(F_{repv}\left(u_{0}\right),F_{repv}\left(v_{0}\right)\right)}^{T}, & if\;k_{0}\notin M_{0}\\ 0, & if\;k_{0}\in M_{0} \end{array}$$ (11)

### Repulsion field and repulsion force of joint limits

The three joint variables of the spray arm have certain a rotation range. Suppose *q*_*i*_ ∈ (*q*_*imin*_, *q*_*imax*_), *i* = 1, 2, 3, and θ is the influence distance of the joint limit position. As a view constraint, the repulsion field of the joint limit is defined as follows.

$$U_{repq}(q)=\sum_{i=1}^{3}U_{repq}(q_{i})$$ (12)

where

$$U_{repq}\left(q_{i}\right)=\{\begin{array}{ll} {\left(q_{i}-\theta -q_{imin}\right)}^{2}\text{ln}\left(\frac{q_{i}-q_{imin}}{\theta }\right), & if\;q_{i}\in \left(q_{imin},q_{imin}+\theta \right)\\ {\left(q_{i}+\theta -q_{imax}\right)}^{2}\text{ln}\left(\frac{q_{imax}-q_{i}}{\theta }\right), & if\;q_{i}\in \left(q_{imax}-\theta ,q_{imax}\right)\\ 0, & if\;q_{i}\in \left[q_{imin}+\theta ,q_{imax}-\theta \right] \end{array}$$

Suppose Γ = {q_i_ ∈ [q_imin_ + θ, q_imax_ − θ], i = 1,2,3}. The repulsion force of the spray arm’s joint limit F_repq_ can be expressed as

$$\{\begin{array}{ll} {\text{L}}_{\text{r}}{\left({\text{F}}_{\text{repq}}\left({\text{q}}_{1}\right),{\text{F}}_{\text{repq}}\left({\text{q}}_{2}\right),{\text{F}}_{\text{repq}}\left({\text{q}}_{3}\right)\right)}^{\text{T}}, & \text{if}\;\text{q}\notin \Gamma \\ 0, & \text{if}\;\text{q}\in \Gamma \end{array}$$ (13)

where

**L**_*r*_——Jacobi matrix of the spray arm

$$F_{repq}\left(q_{i}\right)=-\nabla U_{repq}\left(q_{i}\right)=\{\begin{array}{ll} 2\left(q_{i}-\theta -q_{imin}\right)\text{ln}\left(\frac{q_{i}-q_{imin}}{\theta }\right)+\frac{{\left(q_{i}-\theta -q_{imin}\right)}^{2}}{q_{i}-q_{imin}}, & if\;q_{i}\in \left(q_{imin},q_{imin}+\theta \right)\\ 2\left(q_{i}+\theta -q_{imax}\right)\text{ln}\left(\frac{q_{imax}-q_{i}}{\theta }\right)-\frac{{\left(q_{i}+\theta -q_{imax}\right)}^{2}}{q_{imax}-q_{i}}, & if\;q_{i}\in \left(q_{imax}-\theta ,q_{imax}\right)\\ 0, & if\;q_{i}\in \left[q_{imin}+\theta ,q_{imax}-\theta \right] \end{array}$$

### Trajectory planning method

According to the gravitational and repulsive forces defined above, the resultant force of the spray arm in Cartesian space should be

$$F(P)=F_{att}(P){+\beta F}_{repv}(P)+{\gamma F}_{repq}(P)$$ (14)

where

*β*, *γ*——scaling factors.

The proportion of each component in F(P) can be changed by adjusting the values of β and γ.

A feasible discrete trajectory for the eye-in-hand camera Γ = {P_k_|k = 0,1, ⋯ n} can be planned in Cartesian space by using F(P) and Eq (15).

$$P_{k+1}=P_{k}+\varepsilon_{k}\frac{F(P)}{\left|F(P)\right|}$$ (15)

where

ε_k_——step size of the kth control cycle

Further, combined with the eye-in-hand camera model, the values $s_{k}^{*}$ of image moment $s={\left[\begin{array}{ccc} \frac{m_{10}}{m_{00}} & \frac{m_{01}}{m_{00}} & k\sqrt{\frac{\mu_{20}+\mu_{02}}{m_{00}}} \end{array}\right]}^{T}$ corresponding to each trajectory point P_k_ can be calculated. That is, a corresponding discrete trajectory in the image space $Χ=\{s_{k}^{*}|k=0,1,\cdots n\}$ is obtained.

### Controller design

The image-based visual servo controller is used to track this trajectory. Suppose that in the *k* th control cycle, the error of the image features is defined as:

$$e=s-s_{k}^{*}$$ (16)

A speed controller is selected to exponentially decrease the error. According to reference [28], the controller can be selected as

$$v_{c}=-\lambda {\hat{L}}_{s}^{+}e+{\hat{L}}_{s}^{+}\dot{e}$$ (17)

where

${\hat{L}}_{s}^{+}$——pseudo-inverse of estimation of image Jacobian matrix ***L***_*s*_.

**Fig 5:**
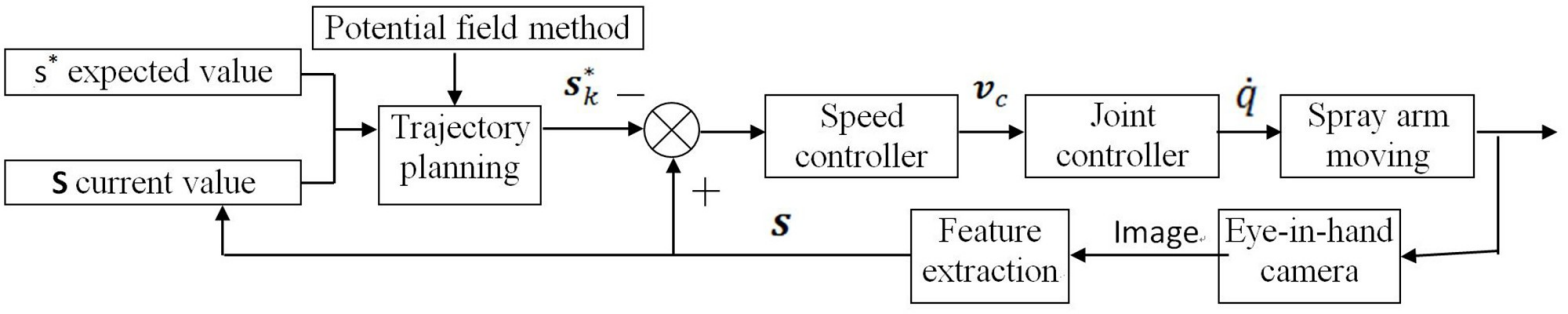
Block diagram of the proposed visual control system.

The visual system block diagram is shown in Fig 5. According to reference [16], the controller can make the system robust to model error and noise disturbance.

## Simulation analysis

**Fig 6:**
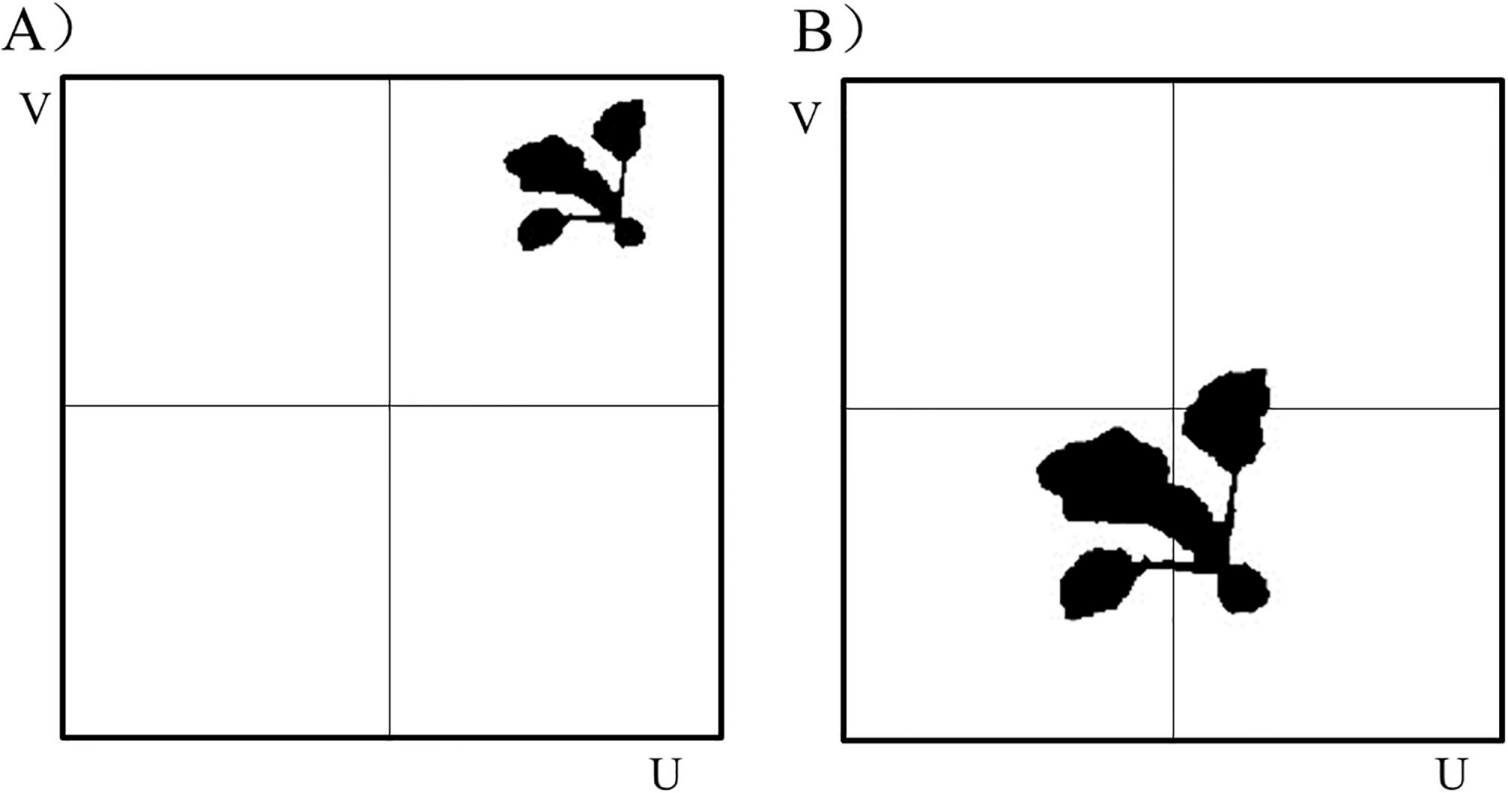
Simulation image. (A) Initial position. (B) Desired position.

The above visual servo motion planning method based on the improved potential field method is validated by simulation in MATLAB software. The camera adopted a perspective projection model with a resolution of 1000 *1000 and a focal length of 10mm. Suppose that the spray robot moves at the speed of *v*_*hx*_ = 0.02m/s, *v*_*hy*_ = 0.3m/s and *v*_*hz*_ = 0m/s. A crop image parallel to the eye-in-hand camera plane, is selected after background segmentation and binarization as the target crop. The projection of the target crop at initial position and the desired spray position are shown in Fig 6. At the desired position, the canopy’s depth of the target crop is Z* = 0.49*m*, and ***S*** = [493 582 262]^*T*^. Suppose that the spray robot stands still, and the target crop moves toward it.

### (1) Effect of adding gravitational field

**Fig 7:**
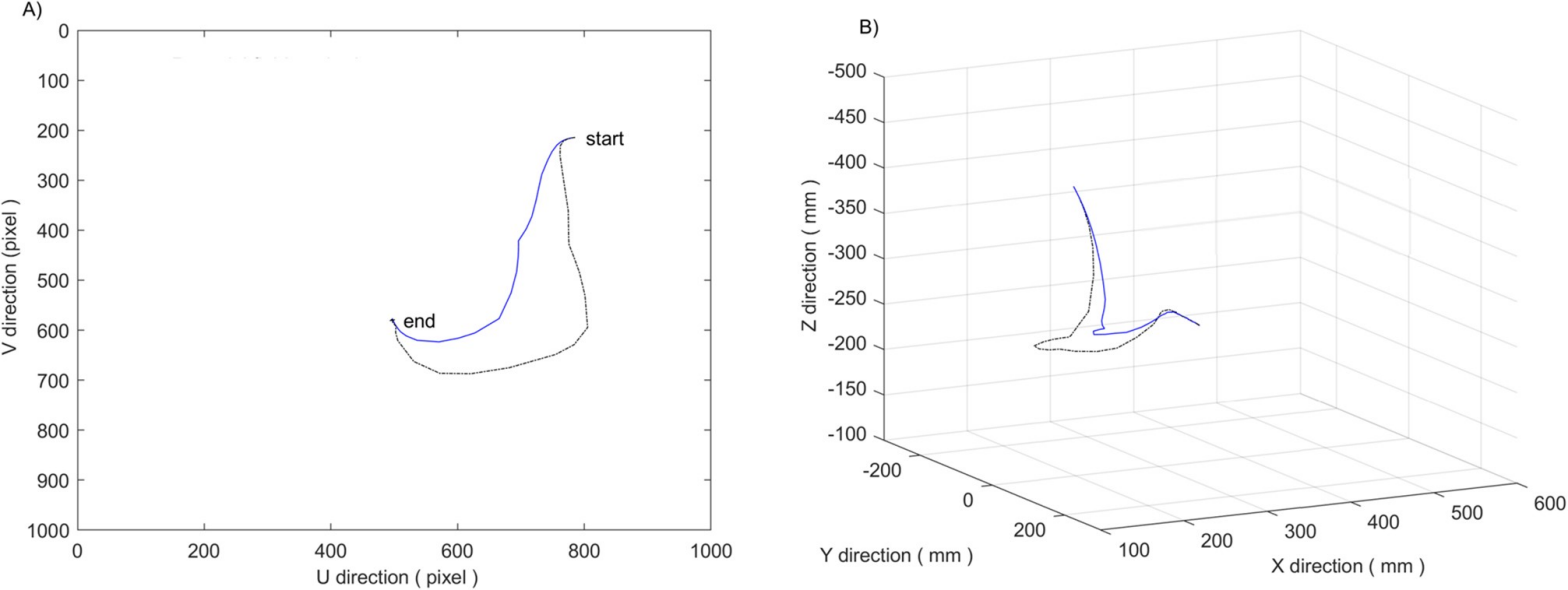
Results of introducing gravitational field. (A) Motion trajectory in image space. (B) Motion trajectory in Cartesian space. Where the simulation result of potential field method is designated with a blue solid line, and the simulation result of image moment method is designated with a black dash-dotted line.

Fig 7 shows a simulation comparison of the planned trajectories between the potential field method with only a gravitational potential term and the image moment method in reference [6]. It can be seen that the nozzle’s trajectory in image space of the former is obviously better than that of the latter, and the nozzle’s trajectory in Cartesian space of the former is shorter and more reasonable than that of the latter.

### (2) Effect of adding field of view constraints

**Fig 8:**
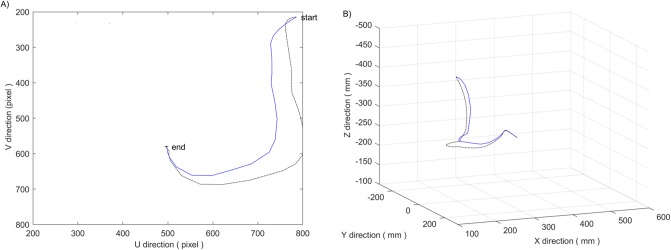
Results of introducing visual field constraints. (A) Motion trajectory in image space. (B) Motion trajectory in Cartesian space. Where the simulation result of potential field method is designated with a blue solid line, and the simulation result of image moment method is designated with a black dash-dotted line.

The field boundary corresponding to the centroid k_0_(u_0_, v_0_) of the crop image is set as u_0_ ∈ [200,800] and v_0_ ∈ [200,800], and the influence distance of the field boundary is set as 100 pixels. Fig 8 shows a simulation comparison of the planned trajectories between the potential field method with only field of view constraints and the image moment method in reference [6]. It can be seen that the latter can not ensure that the target crop always appears in the field of vision, which may lead to the failure of the servo task, and the former can avoid the problem.

### (3) Effect of adding joint limit constraints

**Fig 9:**
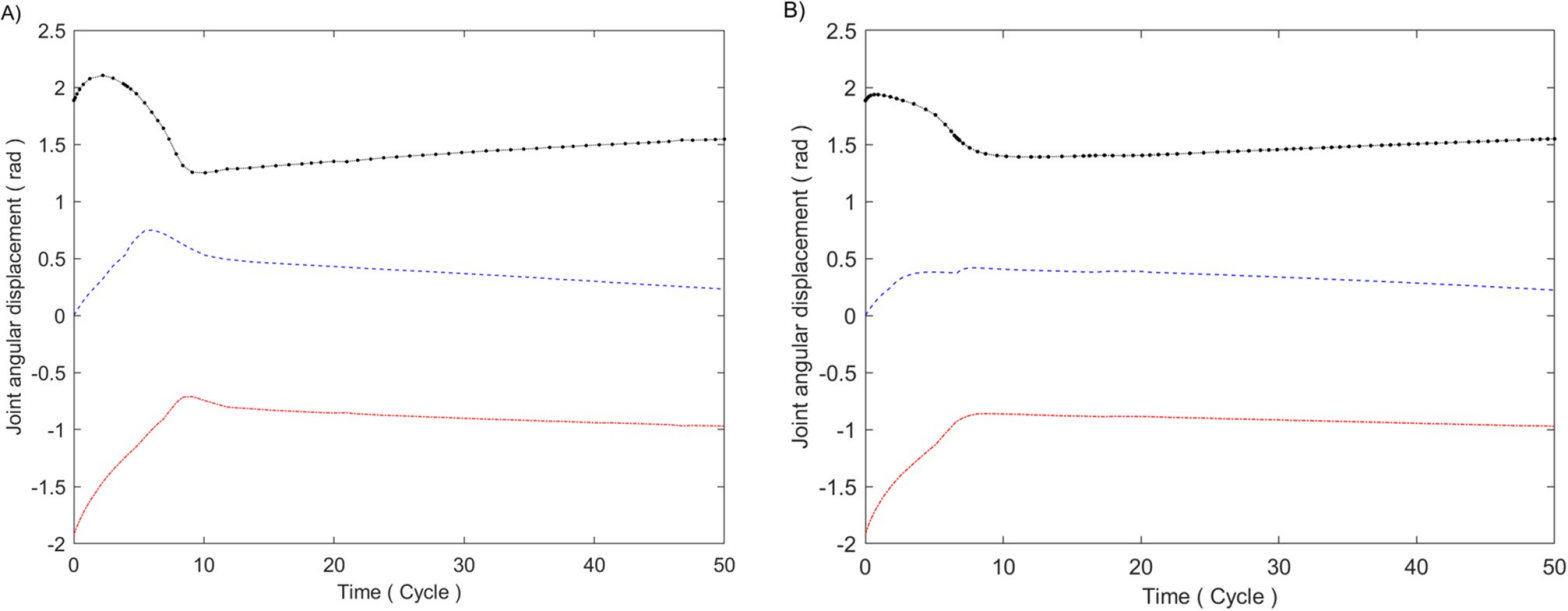
Results of introducing joint limit position constraints. (A) Image moment method. (B) Potential field method. Where the trajectory of the waist is designated with a blue dotted line, the trajectory of the upper arm is designated with a red dash-dotted line, and the trajectory of the lower arm is designated with a black pecked line.

The joint angle variation range of the waist, upper arm and lower arm joints are set as q_1_ ∈ (−1.7, 0.5)rad, q_2_ ∈ (−2.0, −0.2)rad and q_3_ ∈ (0.9,2.0)rad respectively. The influence distance of the joint limit position is set to 0.3 rad. Fig 9 is a simulation comparison of the planned trajectories between the potential field method with only joint limit constraints and the image moment method in reference [6]. It can be seen that in this case, the trajectory planned by the latter makes the joints of both the waist and the lower arm exceed the setting range, leading to the failure of the servo task, while the trajectory planned by the former can avoid the problem.

### (4) Comprehensive effect of the improved potential field method

**Fig 10:**
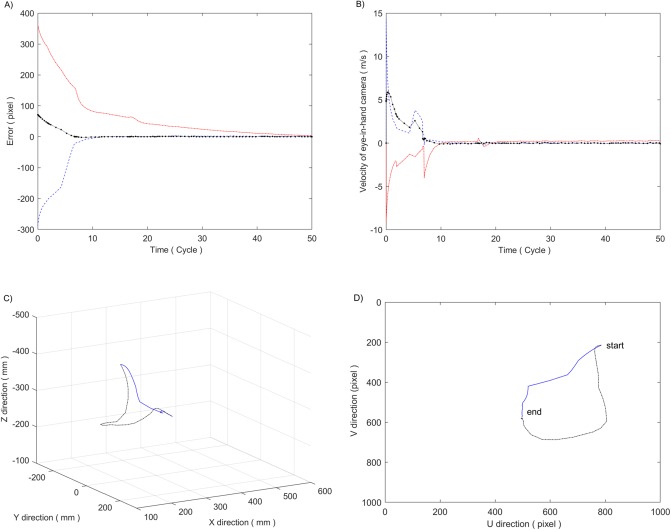
Results of the improved potential field method. (A) Error tracking curve. Where the error tracking curve of moment feature 1 is designated with a blue dotted line, the error tracking curve of moment feature 2 is designated with a red dash-dotted line, and the error tracking curve of moment feature 3 is designated with a black pecked line. (B) Velocity of the eye-in-hand camera. Where the velocity in X direction is designated with a blue dotted line, the velocity in Y direction is designated with a red dash-dotted line, and the velocity in Z direction is designated with a black pecked line. (C) Motion trajectory in Cartesian space. Where the simulation result of potential field method is designated with a blue solid line, and the simulation result of image moment method is designated with a black dash-dotted line. (D) Motion trajectory in image space. Where the simulation result of potential field method is designated with a blue solid line, and the simulation result of image moment method is designated with a black dash-dotted line.

The simulation results of the improved potential field method considering all the gravitational and repulsive terms are shown in Fig 10. Fig 10A is an error tracking curve of three moment features. It can be seen that all the moment features steadily converge and the target process lasts approximately 13 visual cycles. Influenced by the translation velocity of the robot in the X and Y directions, the errors of the three moment features in the tracking process are approximately −3 pixels, −4 pixels and 1 pixel, corresponding to position errors at the desired positions of −1.5mm, −2mm and 0.5mm, respectively. Fig 10B is velocity of the eye-in-hand camera (or nozzle) in the X, Y and Z directions, which converge to −0.02*m*/s, −0.3*m*/s and 0*m*/s, respectively, ensuring the target accuracy in the tracking spray stage. Fig 10C and 10D show the simulation comparison of the planned trajectories for the eye-in-hand camera between the potential field method and the image moment method in reference [6] in Cartesian space and image space, respectively. It can be seen that the trajectories in image space and Cartesian space of the former are obviously better than those of the latter.

## Experimental verification

**Fig 11:**
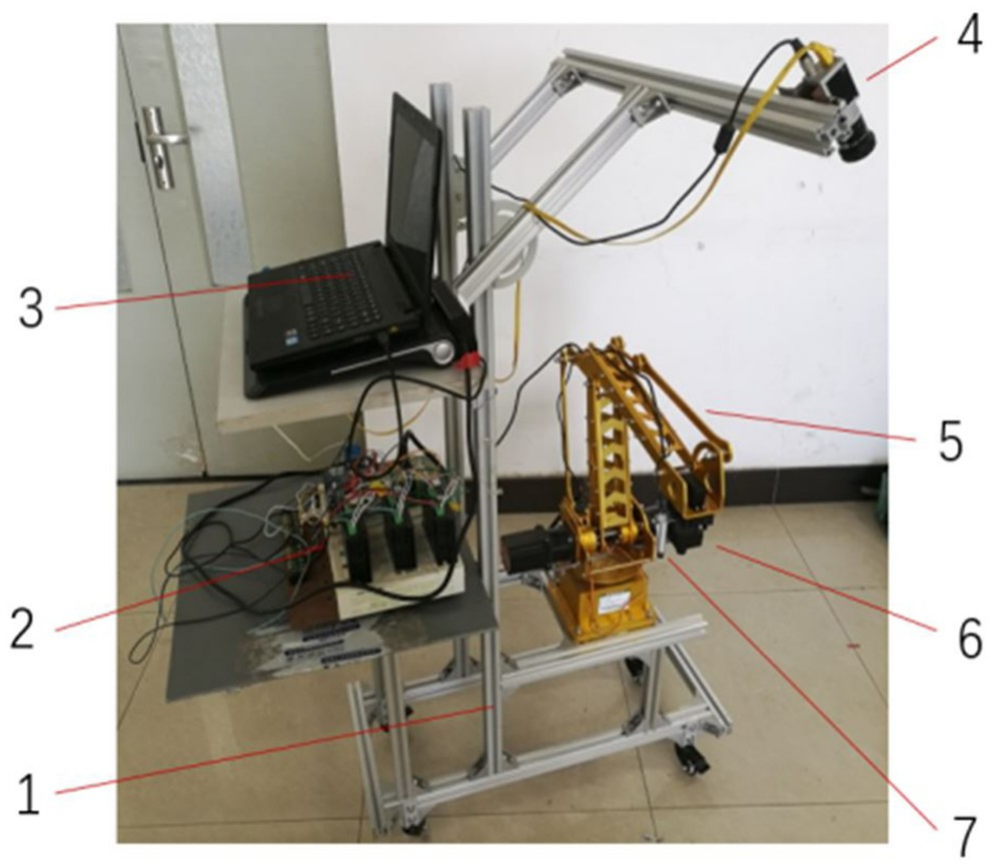
Experimental prototype. 1. Movement platform 2. Controller, driver, etc. 3. PC 4. Scene camera. 5. Spray arm 6. Eye-in-hand camera 7. Laser.

Based on the preceding studies (reference [6]), an improved prototype (Fig 11) is built to test the effectiveness of the above motion planning method. The prototype used a ZUTO460-BRG type 4-axis manipulator as the sprayer arm, a Basler acA1300 industrial camera (resolution 1296*966 pixels) as the scene camera and an OV7725 chip network camera (resolution 640*480 pixels) as the eye-in-hand camera. Moreover, a KYD650N5 controllable laser lamp is used instead of a nozzle to measure the target error conveniently in the experiments.

The pavement of the greenhouse is relatively flat, and random vibration caused by unevenness is generally small. To facilitate the measurement of the target error in the experiment process, prototype experiments are carried out in a controllable laboratory environment.

**Fig 12:**
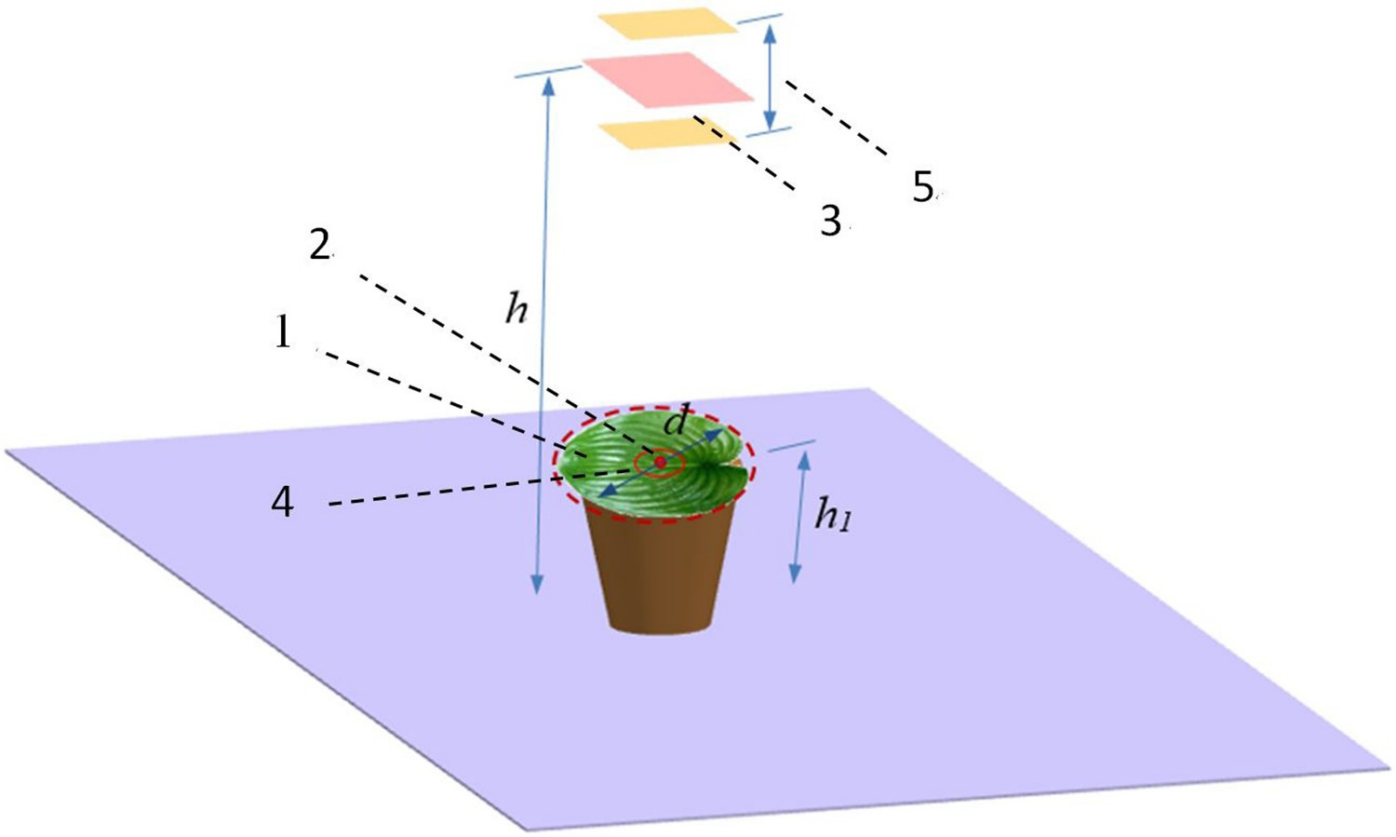
Schematic diagram of target and tracking effects. 1. Blade 2. Centroid (horizontal expected position) 3. Vertical desired position. 4,5. Horizontal and vertical range of effective target tracking.

Ten hawthorn leaves are selected as experiment objects. Before the experiment, the centroid for each leaf was marked, and the expected off-ground height of the laser lamp was estimated when targeting according to *h* = *h*_1_ + 2.8*d* (where *h*_1_— off-ground height of leaf, *d*—diameter of the surrounding circle, the center of which is the centroid of the leaf), as shown in Fig 12. In the experiment, the laser lamp would be lit during the simulated tracking spray process.

The deviation of the laser lamp’s spot relative to the centroid mark during the tracking spray process is taken as the horizontal error *err_h*, and the maximum deviation of the laser lamp’s height relative to its desired height is taken as the vertical error *err_v*.

To quantitatively evaluate the tracking spray’s effect, it is stipulated that the horizontal relative error Re_h = |err_h|/d*100%, the vertical relative error Re_h = |err_h|/h*100%, and the tracking spray effect is divided into two kinds: effective tracking spray (Re_h ≤ 10% and Re_h ≤ 5%) and ineffective tracking spray (Re_h > 10% or Re_v > 5%).

Image processing and feature extraction are carried out using the method used in reference [6]. The method determines the expected value of the image moment is as follows: manually control the spray arm to move the nozzle into its desired spray location, and record the value of $S=\{\frac{m_{10}}{m_{00}},\frac{m_{01}}{m_{00}},k\sqrt{\frac{\mu_{20}+\mu_{02}}{m_{00}}}\}$ at the moment. The process is repeated five times, the average value is taken as the expected value, and the experiment results showed that the expected value of ***S*** is {237, 189, 202}.

**Fig 13:**
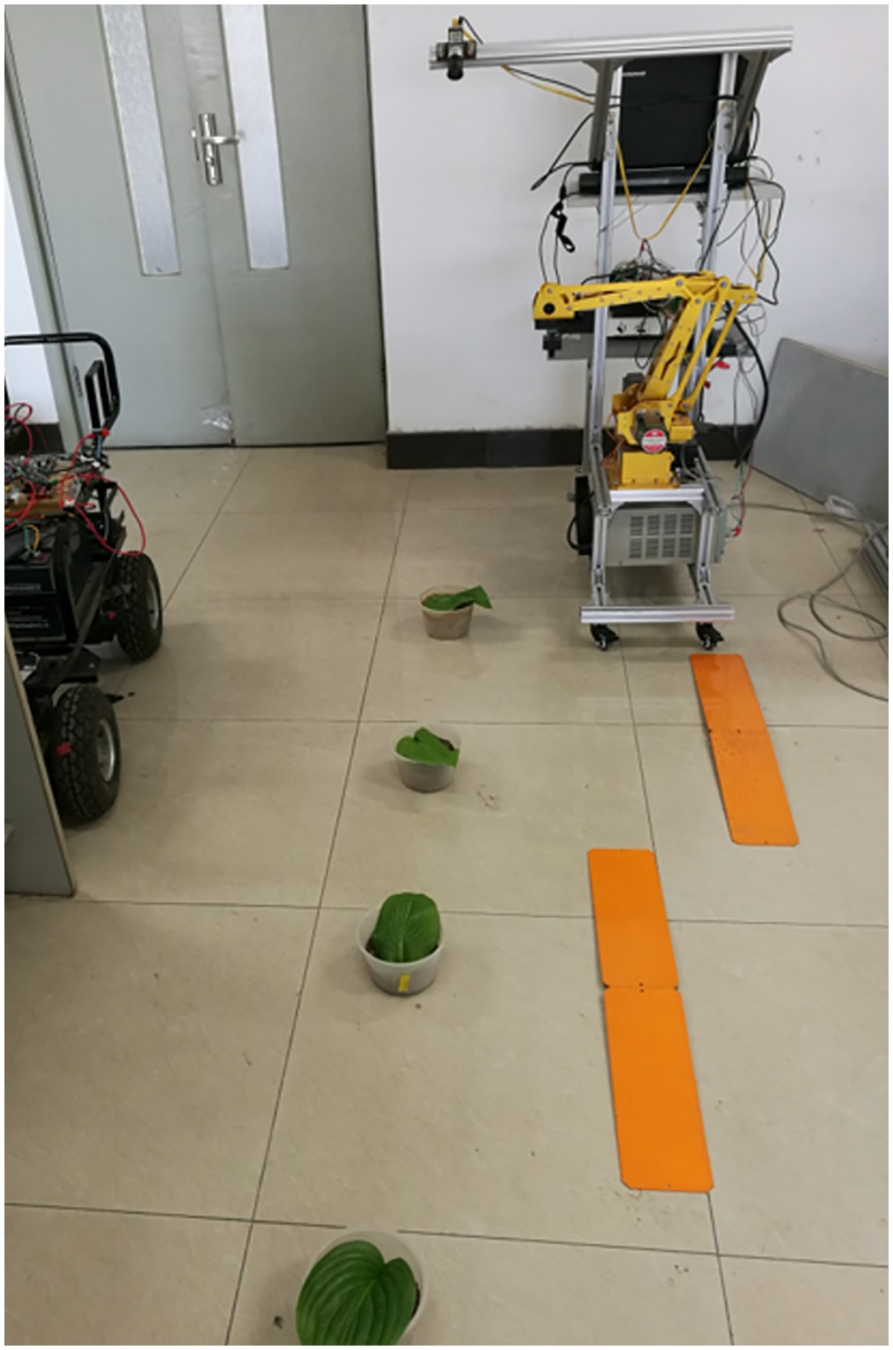
Simulated target experiment.

During the experiments, the leaves are randomly placed with spacing of approximately 30~50*mm* in the X direction and 400~600*mm* in the Y direction, and several obstacles are set in the path of the prototype to simulate the unevenness of greenhouse pavement. The prototype is controlled to pass the obstacles and simulate target spraying on those leaves by two speeds of 100~150mm/s and 250~300mm/s, and each spray time is set to 0.5s, as shown in Fig 13.

To compare the control effect of the improved potential field method with the image moment method of reference [6], ten experiments were carried out for each of the two methods. During the experiments, it was found that the value of Re_h was usually less than 4% of that expected for very special cases, such as the incorrect identification of leaves, while Re_h was always greater than 10% in these cases. Therefore, the factor of whether or not Re_h was greater than 10%, was only used to judge the tracking spray effectiveness in the experiment. For ease of judgment, a marked circle was drawn on each leaf with the centroid as the center and a radius of 0.1d. If the laser lamp’s spot was always in the marked circle during simulated spraying, the tracking spray was judged to be effective; otherwise, the tracking spray was ineffective.

The statistical results of effective and ineffective tracking spray in various cases are shown in Table 1. In the table, the effective tracking spray rate = the times of effective tracking spray / the total times of tracking spray * 100%, and the ineffective tracking spray rate = the times of ineffective tracking spray / the total times of tracking spray * 100%.

**Table 1:** Statistical table of target and tracking effect.

method	velocity (mm/s)	effective tracking spray rate (%)	ineffective tracking spray rate (%)
Image moment method	100–150	82.7	17.3
	250–300	50.2	49.8
Improved potential field method	100–150	94.5	5.5
	250–300	80.3	19.7

The prototype test shows that the improved potential field method has a better tracking control effect than the image moment method in reference [6], and its advantages are more obvious with the increase of the velocity of the prototype. However, the former has higher calculation costs and increases the sampling period by approximately 21%, which is disadvantage and restricts the improvement of the prototype’s velocity and target tracking accuracy. In addition, some factors, such as the model error of the prototype system and the deviation between the actual and ideal attitudes of the leaves are still important factors affecting the target tracking accuracy.

## Conclusion

To further improve the motion control of the spray arm during the processes of targeting and tracking, and based on previous research about visual tracking methods that used image moments and a hybrid vision structure with a single scene camera and a single (or multi) eye-in-hand camera, a novel algorithm for motion planning and target control is proposed. This novel algorithm is based on an improved potential field algorithm and introduces velocity potential field, field of view constraints and joint position limit constraint based on the traditional artificial potential field. Specifically, the velocity potential field is introduced to achieve stable tracking by making the target state of the spray arm move at the same velocity as the crop (relative velocity). The field of view constraint is introduced to prevent the target crop escape from escaping from the field view of the eye-in-hand camera and to ensure the efficiency of the visual servo control. Joint position limit constraints are introduced to ensure that the planned trajectories of the joints are always within an allowable range. The simulation and experimental results show that the proposed method has a higher tracking accuracy, a better planned path and a higher robustness than the servo controller based on image moments. Future work will extend the motion planning algorithm of the manipulator to other platforms, such as logistics intelligent sorting systems.

## Supporting information

S1 Fig Flow chart of simulation analysis. (TIF)

S2 Fig Structure diagram of the prototype software system. Visual servo control system adopts master-slave control mode. The host computer is a PC, which is responsible for background segmentation and feature extraction of crop images, calculation of spray operation point’s position, kinematics calculation of the spray arm, trajectory planning, system management and logic control, etc. PC sends control instructions to the slave computer through serial port. The lower computer adopts an advanced DSP, which is responsible for acquiring and processing the signals of photoelectric switch, driving motors according to the instructions of PC, real-time control on the spray arm, and feedback the running state to PC through serial port.
(TIF)

S3 Fig Flowchart diagram of the prototype software system. (TIF)

S1 File Estimation method of crop position and platform speed based on scene camera image. (DOCX)

S2 File Pictures of experiment scene. (DOCX)
