# Supplementary figures and images for: Research on motion planning for an indoor spray arm based on an improved potential field method
Source: PLoS One. 2020 Jan 10;15(1):e0226912. doi: 10.1371/journal.pone.0226912 (PMC6953814; doi:10.1371/journal.pone.0226912)

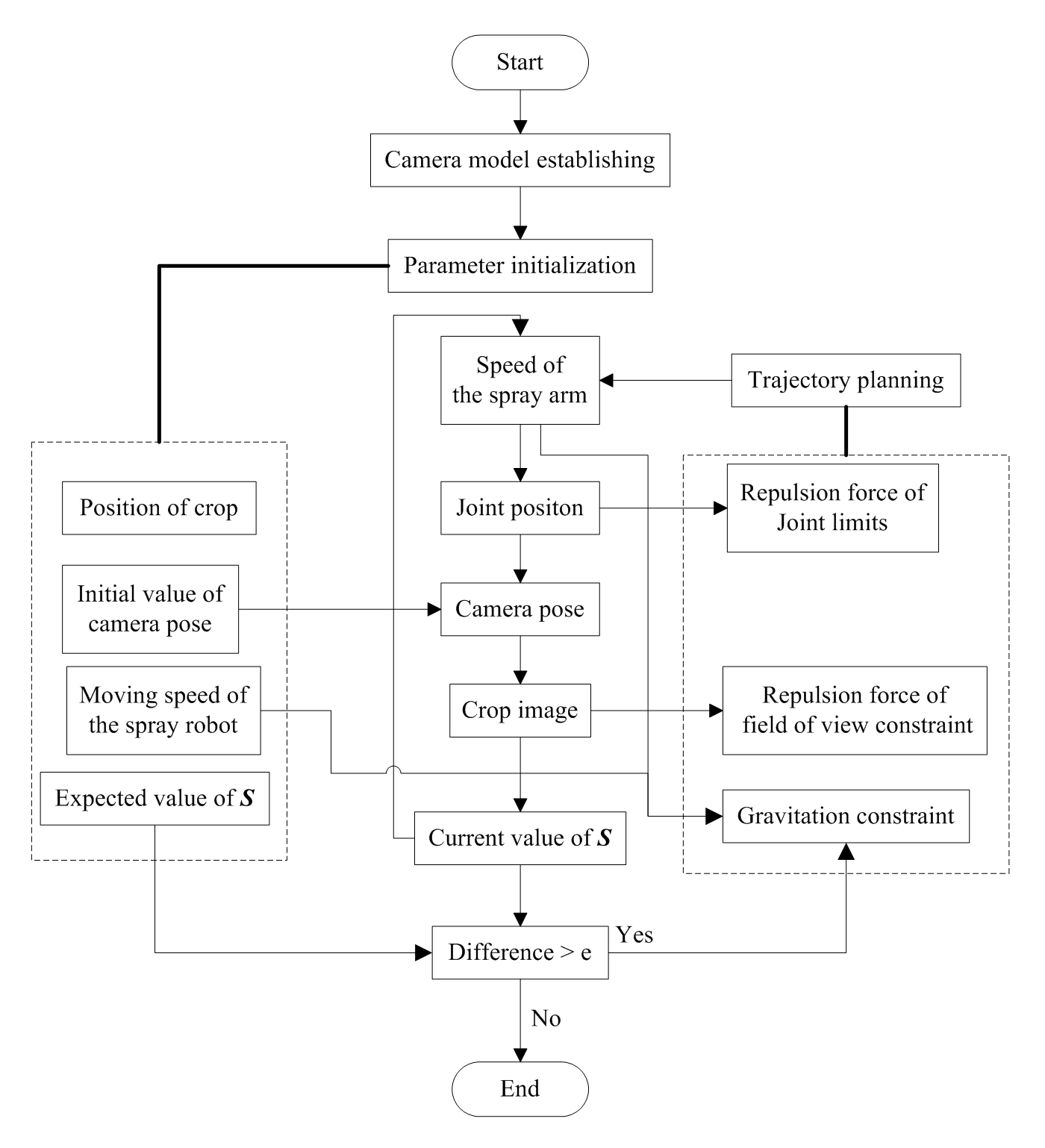

Supplement: S1 Fig — (TIF) [file pone.0226912.s001.tif]

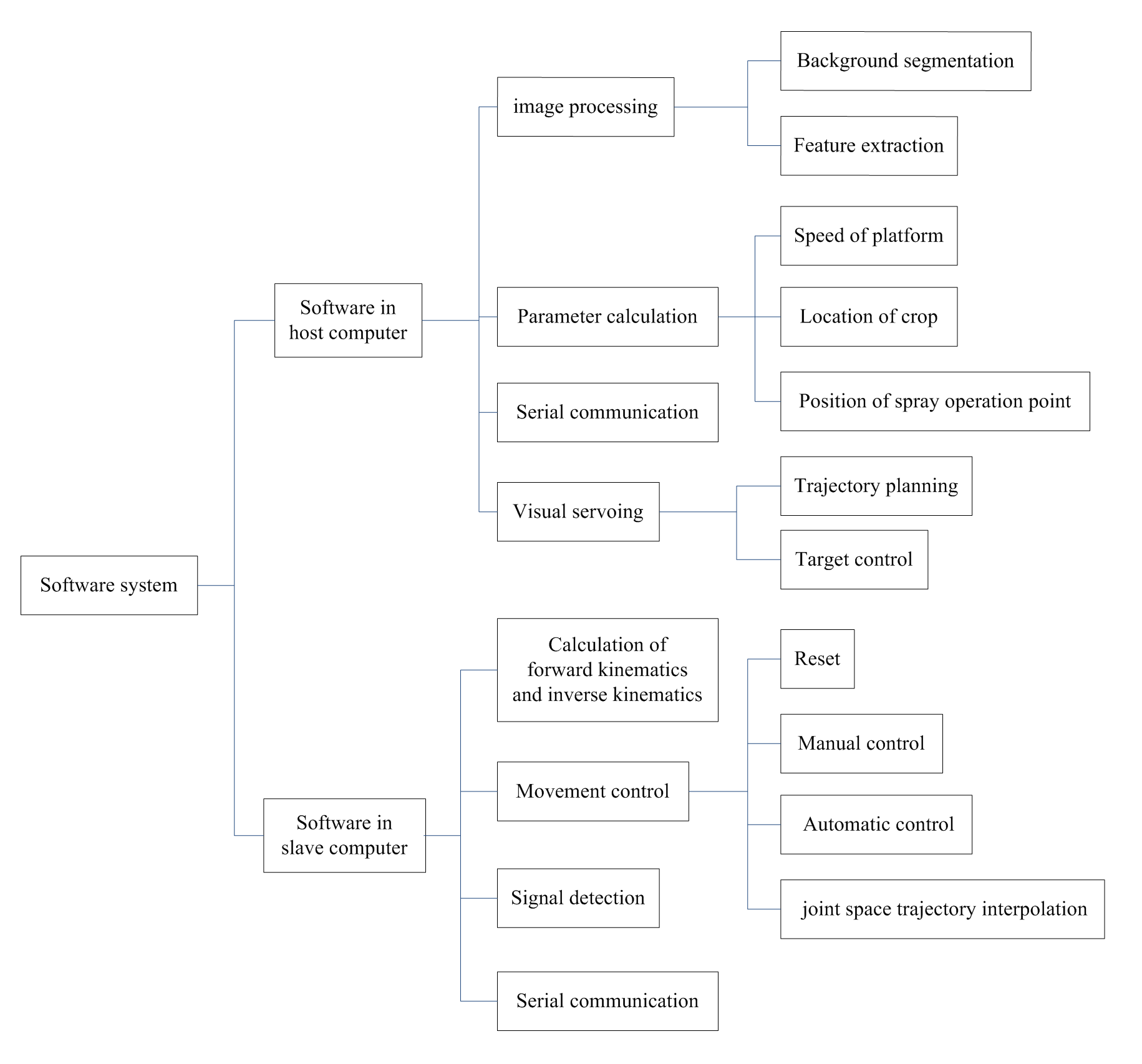

Supplement: S2 Fig — Visual servo control system adopts master-slave control mode. The host computer is a PC, which is responsible for background segmentation and feature extraction of crop images, calculation of spray operation point’s position, kinematics calculation of the spray arm, trajectory planning, system management and logic control, etc. PC sends control instructions to the slave computer through serial port. The lower computer adopts an advanced DSP, which is responsible for acquiring and processing the signals of photoelectric switch, driving motors according to the instructions of PC, real-time control on the spray arm, and feedback the running state to PC through serial port. (TIF) [file pone.0226912.s002.tif]

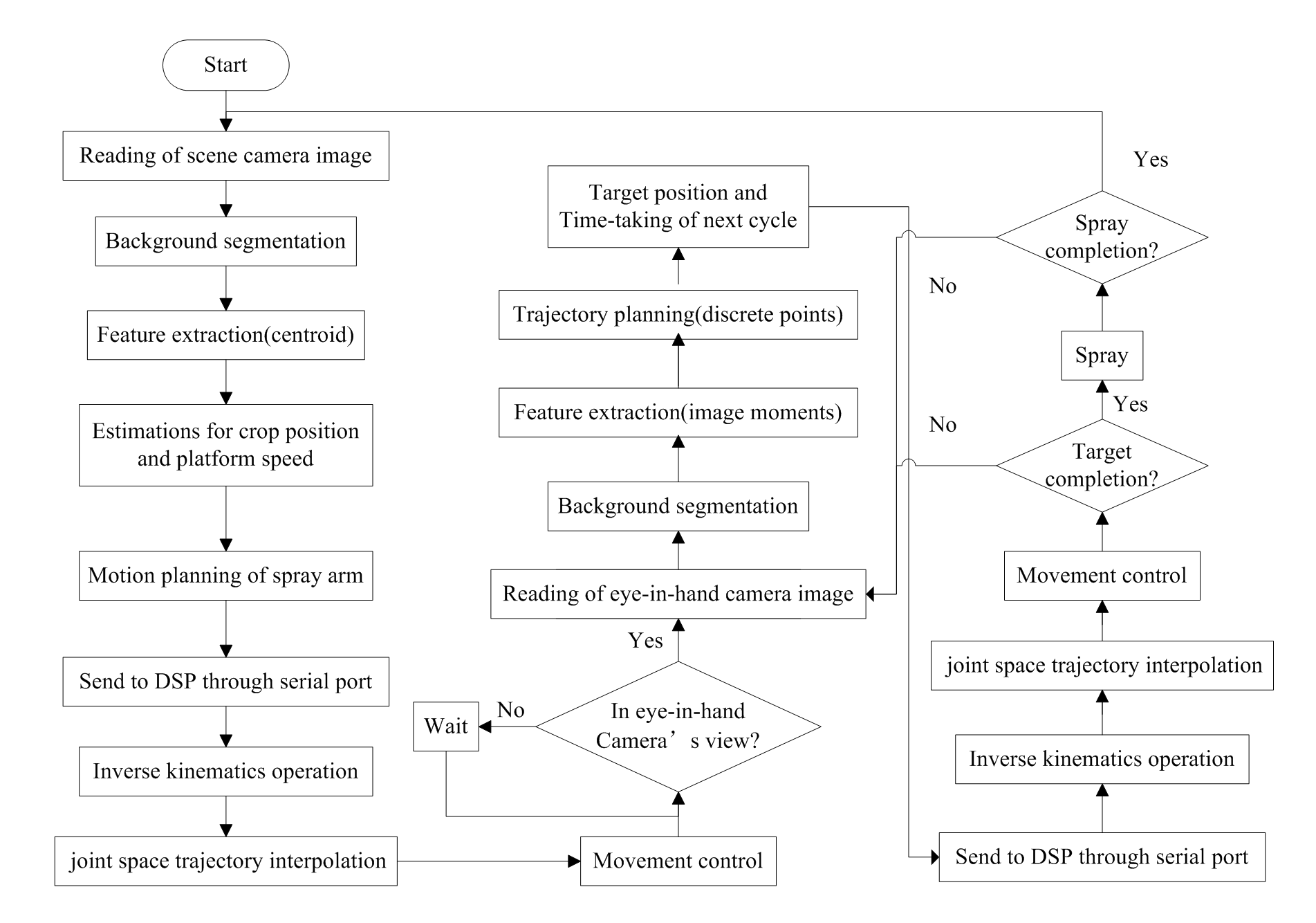

Supplement: S3 Fig — (TIF) [file pone.0226912.s003.tif]
