# Supplementary material for: Research on motion planning for an indoor spray arm based on an improved potential field method
Source: PLoS One. 2020 Jan 10;15(1):e0226912. doi: 10.1371/journal.pone.0226912 (PMC6953814; doi:10.1371/journal.pone.0226912)
Supplement: S1 File — (DOCX) [file pone.0226912.s004.docx]

**Estimation Method of Crop Position and Platform Speed Based on Scene Camera Image**

Scene camera is mounted on robot body, so the transformation matrix between their reference frames ${{}^{r}M}_{c}$ is fixed, which can be derived by Zhang calibration algorithm [1], and we won’t repeat it here. Suppose ${{}^{r}M}_{c}=\left[ \begin{matrix} {{}^{r}R}_{c} & {{}^{r}T}_{c} \\ 0 & 1 \end{matrix} \right]$, then the coordinates of origin of scene camera reference frame $O_{c}$ in robot reference frame is $\left[ O_{cx} O_{cy} O_{cz} 1 \right]^{T}=\left[ {{}^{r}T}_{c} 1 \right]^{T}$。

Assume that the height of crops is equal, and the canopys are within $Z=h$ plane of robot reference frame. Let $Q$ denotes the canopy centroid of some crop, and the image coordinates of its projection point $q$ in scene camera are $\left[ u v \right]^{T}$, then the homogeneous coordinates of $q$ in scene camera reference frame are $\left[ u v f 1 \right]^{T}$, and the homogeneous coordinates of which in robot reference frame are $\left[ q_{x} q_{y} q_{z} 1 \right]^{T}={{}^{r}M}_{c}\cdot\left[ u v f 1 \right]^{T}$, Where $f$ denotes the focal length of scene camera.

According to projection geometrical relationship, $Q$ is the intersection point between $Z=h$ plane and extension line of $O_{c}$ and $q$. Therefore, the coordinates of $Q$in robot reference frame can be expressed as

$\left[ \begin{matrix} Q_{x} \\ Q_{y} \\ Q_{z} \end{matrix} \right]=k\left[ \begin{matrix} q_{x}-O_{cx} \\ q_{y}-O_{cy} \\ 0 \end{matrix} \right]+\left[ \begin{matrix} O_{cx} \\ O_{cy} \\ h \end{matrix} \right]$ （1）

Where, $k=\frac{(h-O_{cz})}{(q_{z}-O_{cz})}$. And ${\begin{matrix} {[Q}_{x} & Q_{y} & Q_{z} \end{matrix}]}^{T}$ is the required location information of target crop.

In addition, let $T$ denotes sampling period, $Q_{k}$ and $Q_{k+1}$ denote the coordinates of $Q$ in robot reference frame at $kT$ and $(k+1)T$ moment, respectively. Then, the movement speed of robot can be approximately expressed as

$V_{h}=\frac{Q_{k}-Q_{k+1}}{T}$ （2）

This study selected a set of coordinates of multiple crop’s canopys at several moments and used least-square method to improve computational accuracy when practically calculate the speed.

# Reference

[1] Z. Y. Zhang, “Flexible camera calibration by viewing a plane from unknown orientations,” in Proceedings of the 7th IEEE International Conference on Computer Vision (ICCV ’99), pp.666–673, Kerkyra, Greece, September 1999.
