# Supplementary material for: Research on motion planning for an indoor spray arm based on an improved potential field method
Source: PLoS One. 2020 Jan 10;15(1):e0226912. doi: 10.1371/journal.pone.0226912 (PMC6953814; doi:10.1371/journal.pone.0226912)
Supplement: S2 File — (DOCX) [file pone.0226912.s005.docx]

Pictures of experiment scene

1、Target experiments of single leaf


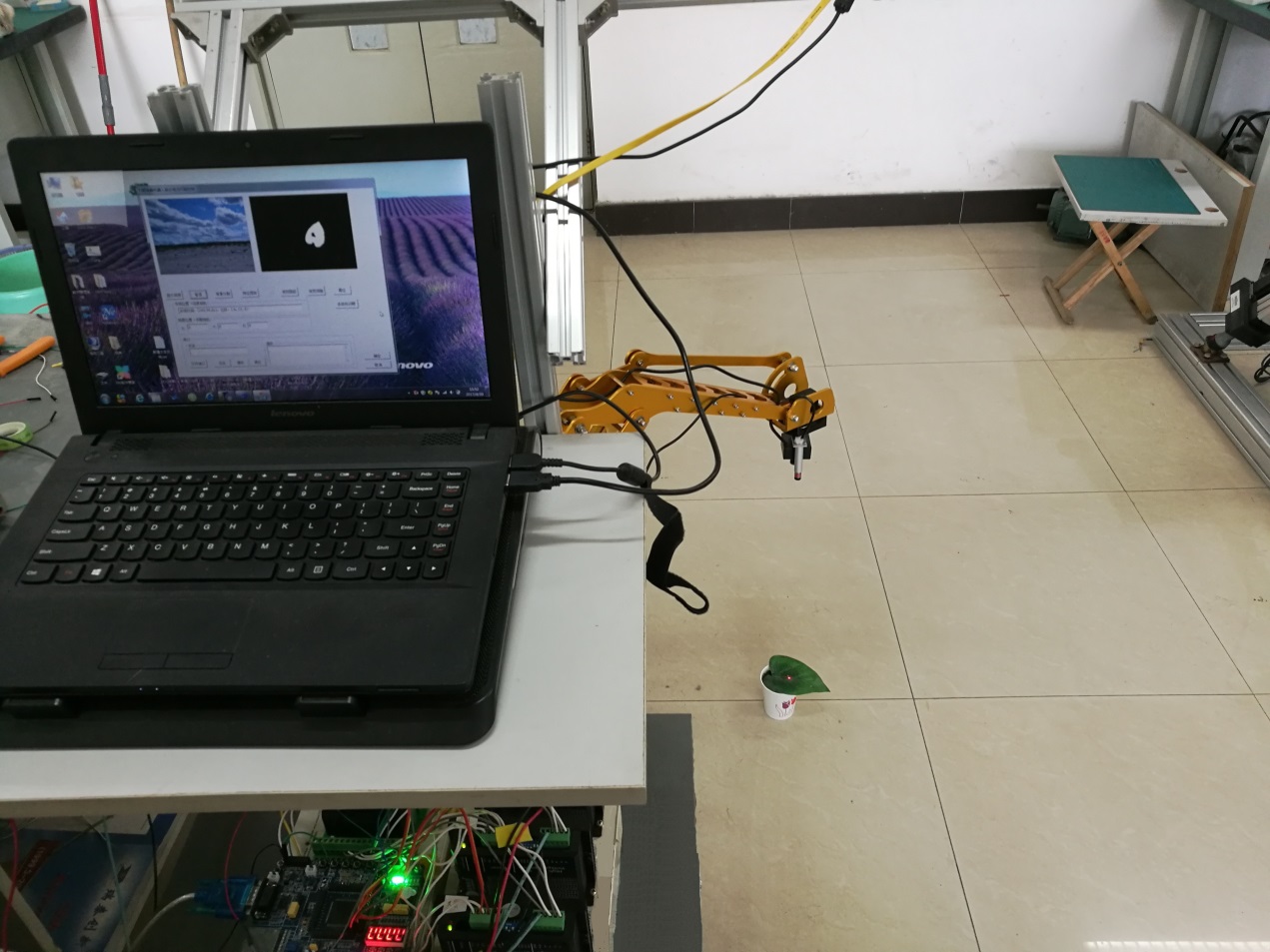


2、Target experiments of multiple leaves


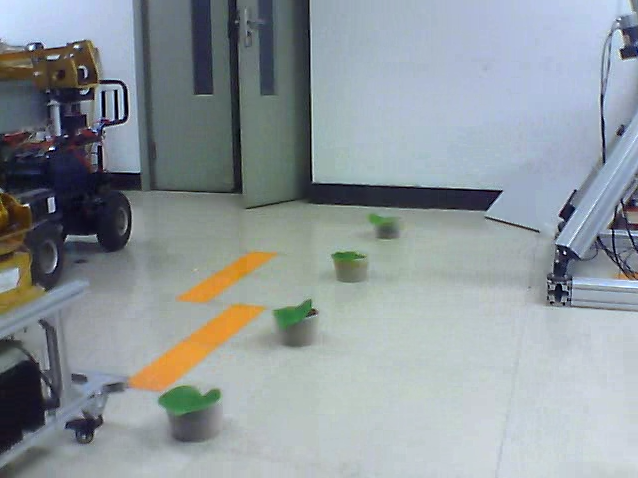

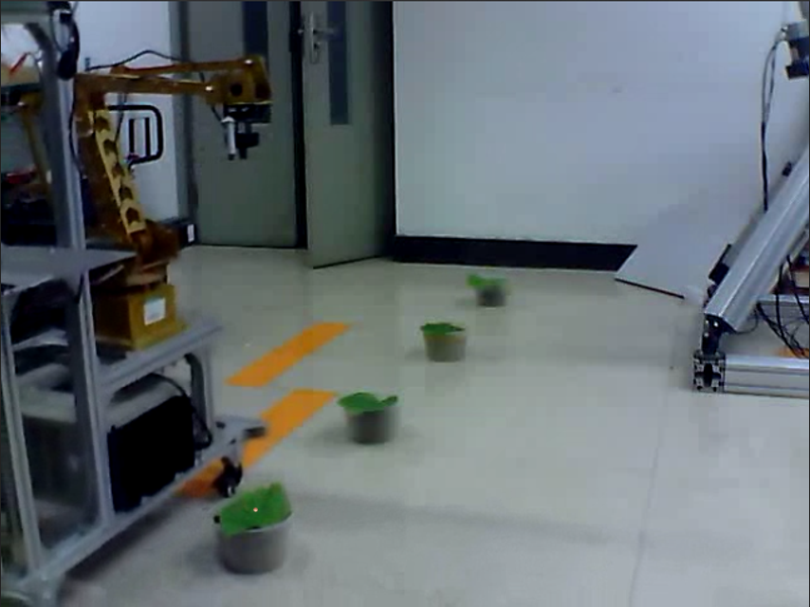


a1) the first leaf (before targeting) a2) the first leaf (targeting)


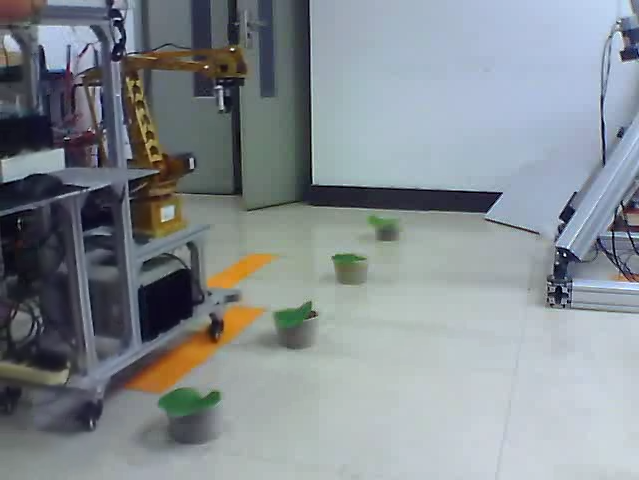

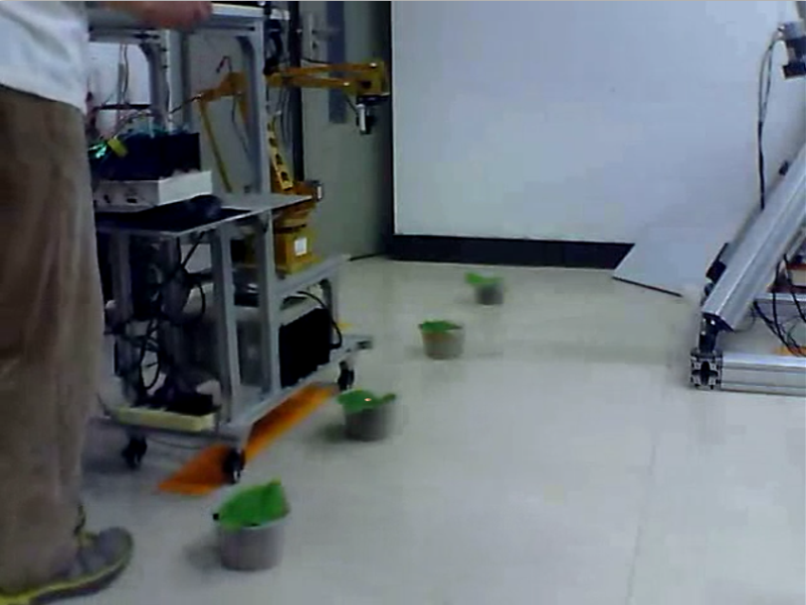


b1) the second leaf (before targeting) b2) the second leaf (targeting)


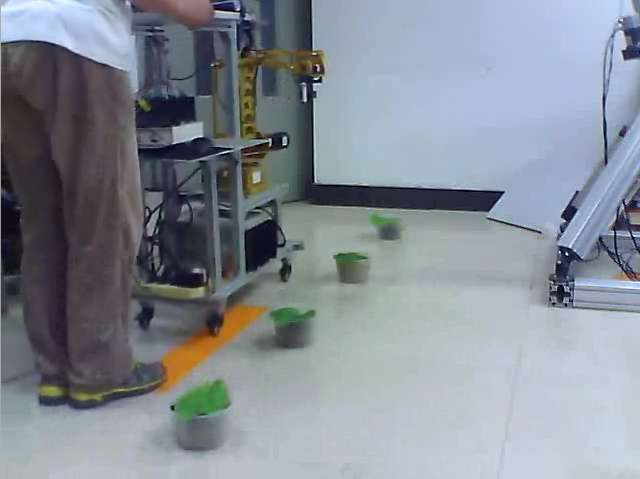

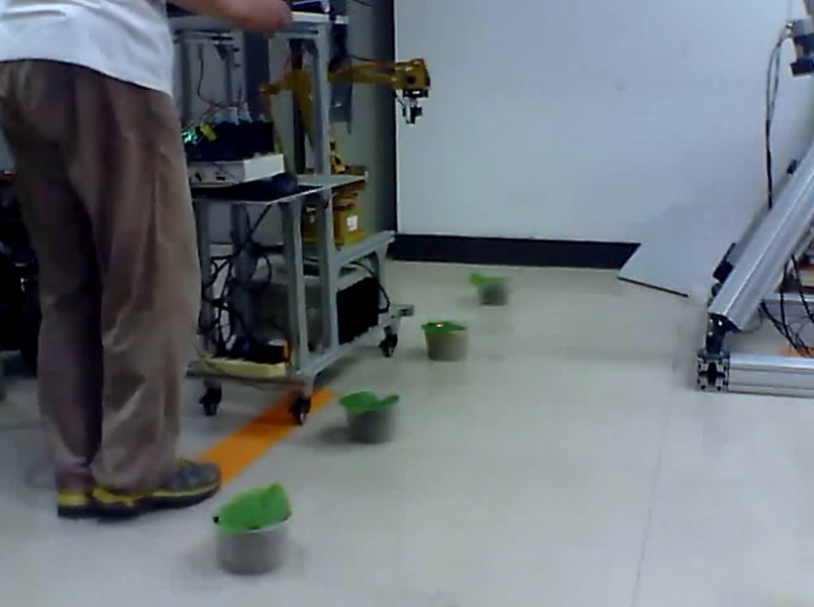


c1) the third leaf (before targeting) c2) the third leaf (targeting)


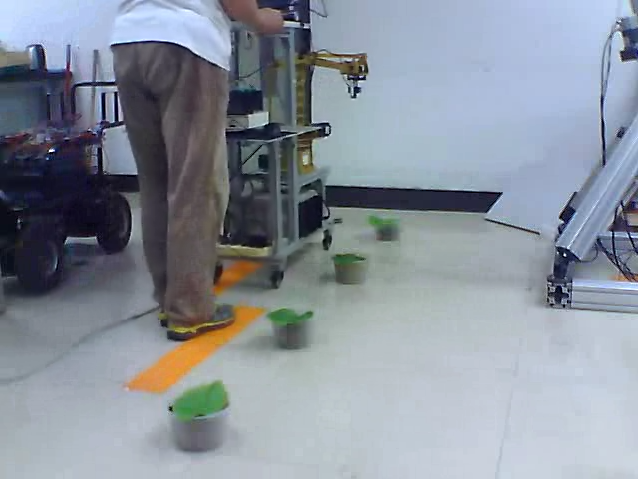

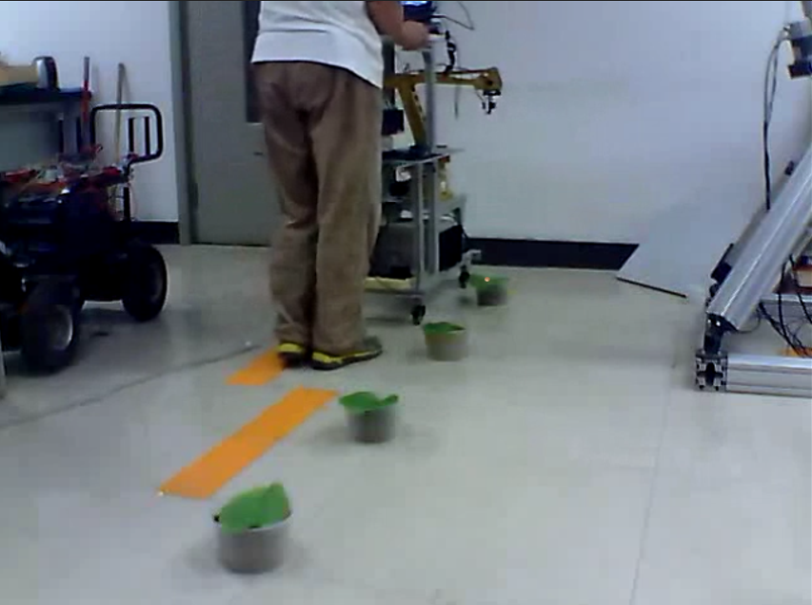


d1) the fourth leaf (before targeting) d2) the fourth leaf (targeting)

3、Target experiments of multiple leaves(Replacement of leaves or obstacles)


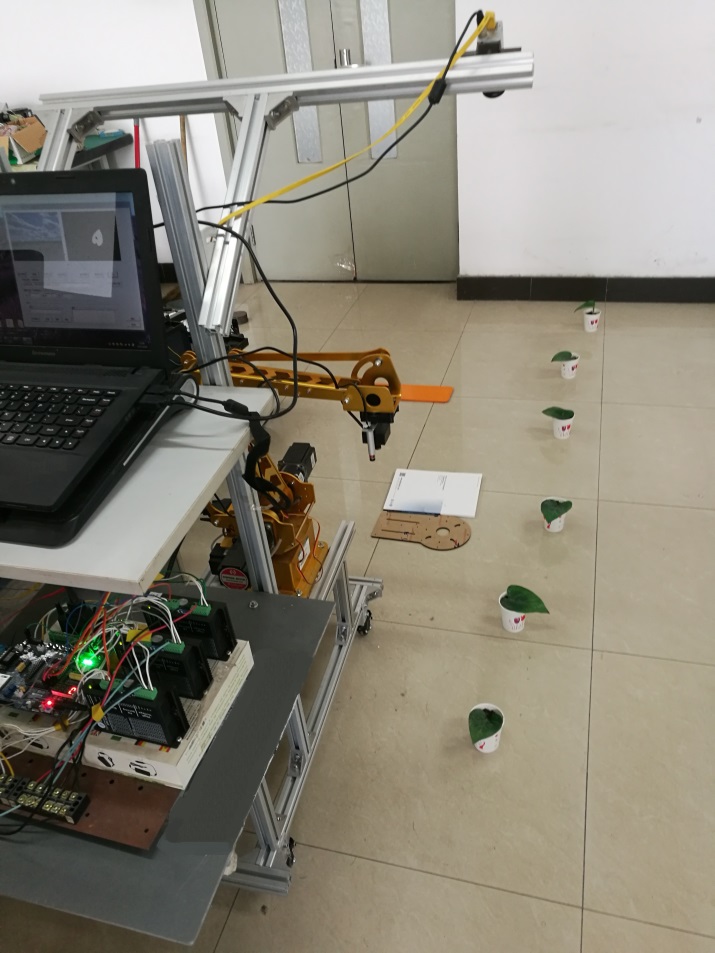

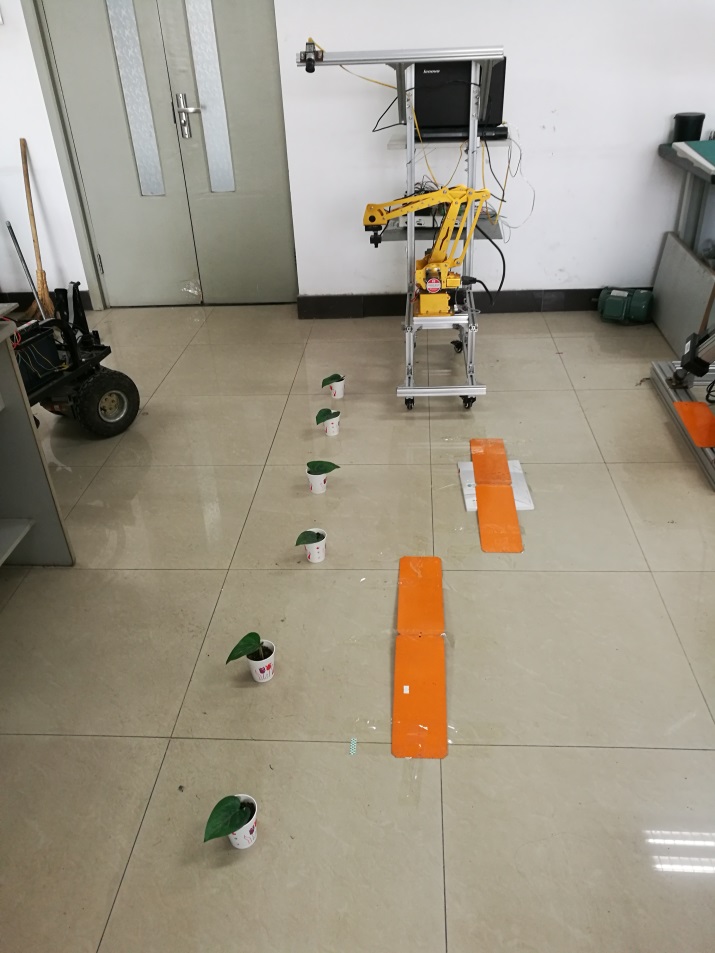


a) b)
